# Supplementary material for: Connecting Colombia’s protected areas: Using a functional approach for tapir species
Source: PLoS One. 2025 May 9;20(5):e0323175. doi: 10.1371/journal.pone.0323175 (PMC12063828; doi:10.1371/journal.pone.0323175)
Supplement: S1 Table — (DOCX) [file pone.0323175.s001.docx]

**Supporting information**

**Supporting Information 1 (S1 Table).** Information on the ecology, behavior, and movements of tapir species reported in the scientific literature.

| **Information on ecology, behaviour and movements** | ***Tapirus bairdii*** | ***Tapirus pinchaque*** | ***Tapirus terrestris*** |
| --- | --- | --- | --- |
| Gestation | 11-13 months | 11-13 months | 11-13 months |
| Sexual maturity | 3 years | 3 years | 3 years |
| Number of offspring | 1 | 1 | 1 |
| Habitats: | Forests, Shrublands, Grasslands and Inland Wetlands | Forests, Scrublands, Grasslands and Inland Wetlands. The only tapir living outside tropical rainforests | Forests, Shrublands, Grasslands and Inland Wetlands |
| Protected Habitat | 255.368 km^2^ | 29.099 km^2^ | 4.923.093 km^2^ |
| Altitudinal range | 0-3.620 | 2.000-4.300 | 0-1.700 |
| Current distribution | 304.884 km^2^ | 49.667 km^2^ | 8.272.254 km^2^ |
| Possibly present | 352.757 km^2^ | 49.667 km^2^ | 3.501.870 km^2^ |
| Locally extinct | 66.827 km^2^ | ^_^ | 1.943.622 km^2^ |
| Home range (km^2^) | 5-30 km^2^ | 2-10 km^2^ | 10-20 km^2^ |
| Dispersion capacity | 10.5 km–10.7 km | 15 km | 8.9 km |
| Number of adult individuals | 3.000 | 2.500 | ^_^ |
| Population | Decreasing | Decreasing | Decreasing |
| IUCN International Threat Category | Endangered (EN) | Endangered (EN) | Vulnerable (VU) |
| National threat category | Endangered (EN) | Endangered (EN) | Vulnerable (VU) |
