# Supplementary material for: Connecting Colombia’s protected areas: Using a functional approach for tapir species
Source: PLoS One. 2025 May 9;20(5):e0323175. doi: 10.1371/journal.pone.0323175 (PMC12063828; doi:10.1371/journal.pone.0323175)
Supplement: S2 Table — (DOCX) [file pone.0323175.s002.docx]

**Supporting information**

**Supporting Information 2 (S2 Table).** Resistance values used for this study.

| **Layer (weight)** | **Category** | **Resistance score** |
| --- | --- | --- |
| Land use and land cover (0.44) | Forest | 0 |
|  | Scrub | 2 |
|  | Water | 4 |
|  | Degraded | 8 |
|  | Barren | 10 |
|  | Agriculture | 49 |
|  | Settlement | 100 |
| Road (0.13) | Absent | 0 |
|  | Other Roads | 50 |
|  | Highways | 100 |
| Population density (0.33) | Absent | 0 |
|  | Low | 30 |
|  | Middle | 60 |
|  | High | 100 |
| Wildfires (0.09) | Absent | 0 |
|  | Present | 100 |
