# Supplementary material for: Connecting Colombia’s protected areas: Using a functional approach for tapir species
Source: PLoS One. 2025 May 9;20(5):e0323175. doi: 10.1371/journal.pone.0323175 (PMC12063828; doi:10.1371/journal.pone.0323175)
Supplement: S4 Fig — (DOCX) [file pone.0323175.s004.docx]

**Supporting information**

**Supporting Information 4 (S4 Figure).** SMD response curves of the *T. bairdii* distribution model.

**
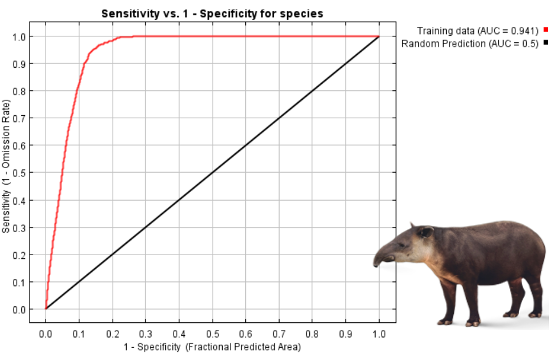
**
