# Supplementary material for: Connecting Colombia’s protected areas: Using a functional approach for tapir species
Source: PLoS One. 2025 May 9;20(5):e0323175. doi: 10.1371/journal.pone.0323175 (PMC12063828; doi:10.1371/journal.pone.0323175)
Supplement: S5 Table — (DOCX) [file pone.0323175.s005.docx]

**Supporting information**

**Supporting Information 5 (S5 Table).** Percent contribution and permutation importance of environmental and human-derived predictors used in the construction of the *T. pinchaque* distribution model.

| **Type of variables** | **Predictors** | **Acronym** | **Percent contribution** | **Permutation importance** |
| --- | --- | --- | --- | --- |
|  | Temperature seaonality | TS | 0.2 | 3 |
|  | Temperature annual range | TAR | 1.9 | 1.5 |
|  | Precipitación of driest month | PDM | 13.6 | 9.8 |
|  | Precipitación of coldest quarter | PCQ | 1.4 | 0.7 |
|  | Isothermality | ISO | 1.2 | 3.2 |
| Habitat Quality | Average  Normalized Difference Vegetation Index - NDVI of the dry season (October to March) for the last 10 years. | NDVI | 0.9 | 0.5 |
|  | Distance to rivers | DRI | 0.2 | 0.1 |
|  | Distance to the forest | DF | 1.4 | 4.2 |
|  | Elevation | ELE | 77.9 | 75.1 |
| Human | Distance to roads | DRO | 0.6 | 0.3 |
|  | Distance to urban centers | DUC | 0.2 | 0.3 |
|  | Human modification | HM | 0.6 | 1.3 |
