# Supplementary material for: Connecting Colombia’s protected areas: Using a functional approach for tapir species
Source: PLoS One. 2025 May 9;20(5):e0323175. doi: 10.1371/journal.pone.0323175 (PMC12063828; doi:10.1371/journal.pone.0323175)
Supplement: S6 Fig — (DOCX) [file pone.0323175.s006.docx]

**Supporting information**

**Supporting Information 6 (S6 Figure).** SMD response curves of the *T. pinchaque* distribution model.

**
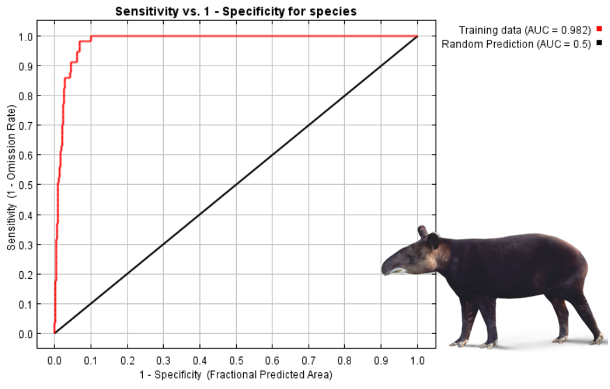
**
