# Supplementary material for: Connecting Colombia’s protected areas: Using a functional approach for tapir species
Source: PLoS One. 2025 May 9;20(5):e0323175. doi: 10.1371/journal.pone.0323175 (PMC12063828; doi:10.1371/journal.pone.0323175)
Supplement: S7 Table — (DOCX) [file pone.0323175.s007.docx]

**Supporting information**

**Supporting Information 7 (S7 Table).** Percent contribution and permutation importance of environmental and human-derived predictors used in the construction of the *T. terrestris* distribution model.

| **Type of variables** | **Predictors** | **Acronym** | **Percent contribution** | **Permutation importance** |
| --- | --- | --- | --- | --- |
| Climatic | Temperature seaonality | TS | 22.8 | 22.2 |
|  | Temperature annual range | TAR | 2.9 | 1.5 |
|  | Precipitación of driest month | PDM | 2.5 | 4.8 |
|  | Precipitación of coldest quarter | PCQ | 16.9 | 8.5 |
|  | Isothermality | ISO | 1.3 | 4.3 |
| Habitat Quality | Average  Normalized Difference Vegetation Index - NDVI of the dry season (October to March) for the last 10 years. | NDVI | 7.9 | 4.9 |
|  | Distance to rivers | DRI | 1.2 | 0.6 |
|  | Distance to the forest | DF | 3.6 | 3.9 |
|  | Elevation | ELE | 16.7 | 20.2 |
| Human | Distance to roads | DRO | 8.5 | 3.9 |
|  | Distance to urban centers | DUC | 9.1 | 7.7 |
|  | Human modification | HM | 6.6 | 17.4 |
