# Supplementary material for: Connecting Colombia’s protected areas: Using a functional approach for tapir species
Source: PLoS One. 2025 May 9;20(5):e0323175. doi: 10.1371/journal.pone.0323175 (PMC12063828; doi:10.1371/journal.pone.0323175)
Supplement: S9 Fig — (A, B) Baird’s tapir, (C, D) Mountain tapir, and (E, F) Lowland tapir. (DOCX) [file pone.0323175.s009.docx]

**Supporting information**

**Supporting Information 9 (S9A-F Figures).** Plots of the metrics are calculated to identify trends and sensitivity to changes in the threshold. (A-B) Baird's tapir, (C-D) Mountain tapir, and (E-F) Lowland tapir.

| **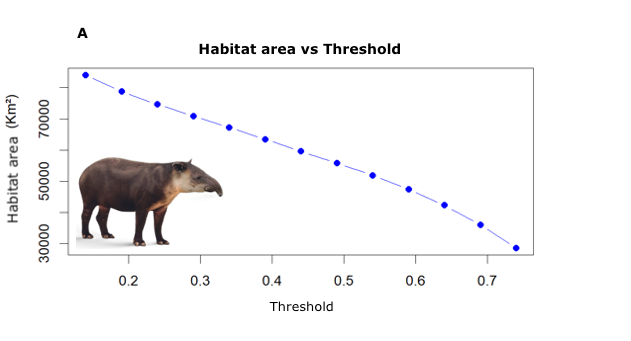** | **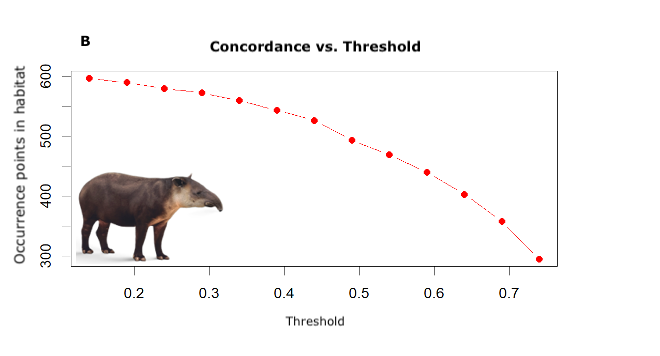** |
| --- | --- |
| **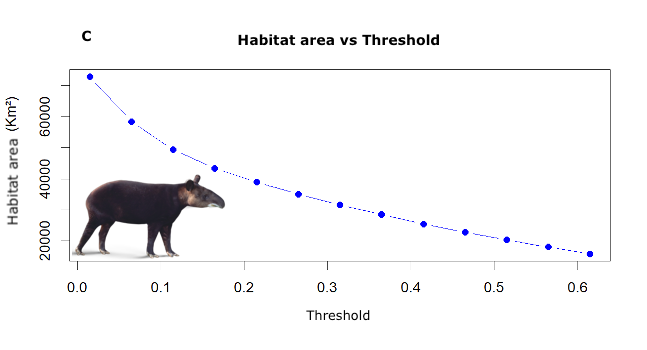** | **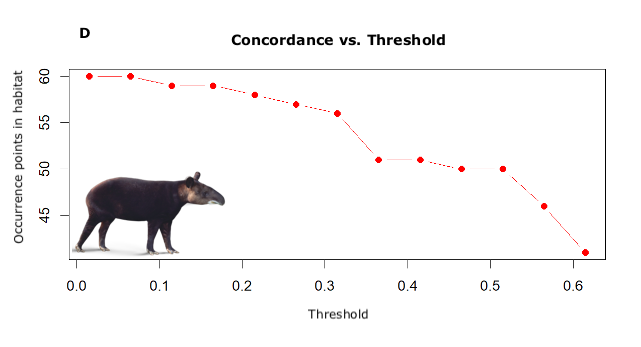** |
| **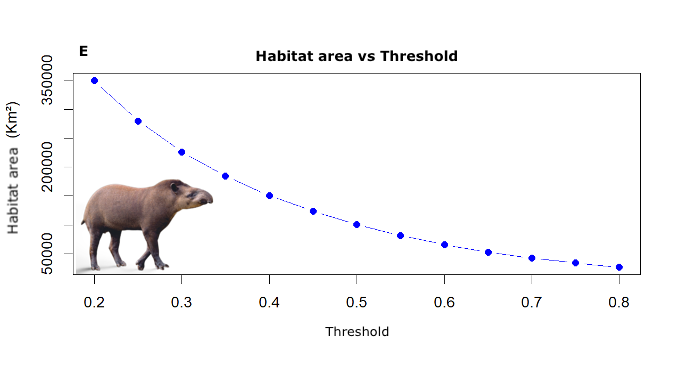** | **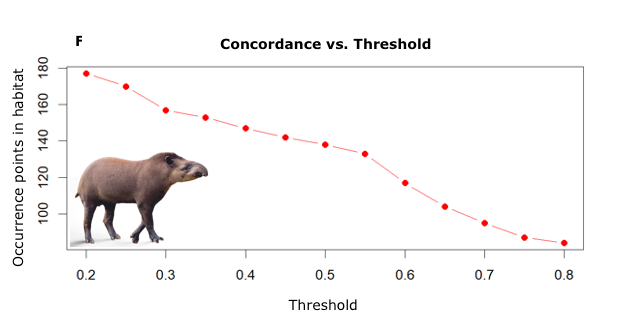** |
