# Supplementary material for: Connecting Colombia’s protected areas: Using a functional approach for tapir species
Source: PLoS One. 2025 May 9;20(5):e0323175. doi: 10.1371/journal.pone.0323175 (PMC12063828; doi:10.1371/journal.pone.0323175)
Supplement: S1 File — This information can be found at the following link. (DOCX) [file pone.0323175.s010.docx]

**Supporting information**

**Supporting Information 10.** The spatial analyses performed for this manuscript were uploaded to the open access platform GitHub. This information can be found at the following link:

https://github.com/jsbarretorunal/Connecting-Colombia-s-protected-areas-Using-a-functional-approach-for-tapir-species/upload/main
